# Mediators of Pruritus in Lichen Planus

**DOI:** 10.1155/2013/941431

**Published:** 2013-07-21

**Authors:** Kalina Welz-Kubiak, Adam Reich

**Affiliations:** Department of Dermatology, Venereology and Allergology, Wroclaw Medical University, Ulica Chalubinskiego 1, 50-368 Wroclaw, Poland

## Abstract

Lichen planus (LP) is an inflammatory mucocutaneous disease, showing a wide variety of clinical subtypes. The classic presentation of LP involves the appearance of polygonal, flat-topped, violaceous papules and plaques with reticulated white lines, termed “Wickham's striae”. Cutaneous lesions tend to be extremely pruritic, and this symptom does not subside after common antipruritic treatment. Moreover, based on our previous pilot study, it could be stated, that itch is the most unpleasant and bothersome symptom of LP for majority of patients suffering from this disease. However, the underlying mechanisms of itch in lichen planus remain still unknown. In addition, there is no study on mediators of this sensation, but taking into account pathogenesis of LP there are some possible mediators implicated to transmit or modulate itch. In pathogenesis of LP important are such mechanisms as apoptosis, autoimmune reaction, and role of stress. With these pathways some, previously described in other diseases, itch mediators such as cytokines, proteases, and opioid system are connected. Whether these mechanisms are involved in pruritus accompanying LP requires further investigation. Limited knowledge of pruritus origin in lichen planus is responsible for the lack of the effective antipruritic treatments. Here, we describe possible mechanisms participating the pathogenesis of pruritus in lichen planus.

## 1. Introduction

Lichen planus (LP) is a chronic inflammatory disease involving both the skin and mucous membranes. This is relatively rare disease, occurring in about 0.5% of general population, with the similar incidence in males and females; the disease rarely develops in children [1].

LP shows a wide variety of clinical manifestations, and numerous subtypes of LP have been described, showing variable lesion configuration and morphology, that is, eruptive LP, inverse LP, mucosal LP, lichen planopilaris, hypertrophic LP, bullous LP, actinic LP, annular atrophic LP, erosive LP, pigmented LP, perforating LP, invisible LP, and others. However, all types of LP have similar histology showing band-like lymphohistiocytic infiltrate at the dermoepidermal junction with vacuolar degeneration of the basal layer of epidermis. Necrotic keratinocytes (civatte bodies or cytoid bodies) are extruded into the papillary dermis. Irregular acanthosis may result in a saw-toothed appearance of dermoepidermal junction. Hyperorthokeratosis may also be seen but is rather considered as a feature of lichenoid drug eruption [2]. 

The classic clinical manifestation of LP involves the presence of polygonal, flat-topped, violaceous papules and plaques with reticulated white lines, termed “Wickham's striae”. It is believed that Wickham's striae result from focal hypertrophy of granular layer of the epidermis. Furthermore, LP lesions may arise as an isomorphic response to trauma (Koebner phenomenon). The disease most commonly affects extremities, particularly the flexural areas of wrists and ankles. Oral involvement is present in about 30–70% of patients with LP. Lesions of oral LP most commonly appear as asymptomatic or tender, white, reticulated patches or plaques (reticulated form) or as painful erosions and ulcers (erosive form). LP of the genitalia most commonly presents with pruritus or hyperalgesia and may lead to vaginal discharge or hemorrhage. 

Importantly, cutaneous lesions of LP tend to be extremely pruritic and this symptom usually does not subside after common antipruritic treatment. Our preliminary studies indicated that pruritus is the most important and bothersome symptom of the disease for the majority of patients suffering from LP [3, 4]. However, to date, the clinical characteristics and pathogenesis of pruritus in LP are nearly completely unknown. 

Itch or pruritus is a cutaneous sensation different from pain. It is evoked by pruritogenic stimuli activating distinct subgroups of dedicated primary afferent C-fibers, including both histamine-sensitive and histamine-insensitive nonnociceptive polymodal nerve fibers, although nociceptive polymodal fibers are also involved to some extend [5–7]. Keratinocytes, leukocytes, mast cells, fibroblasts, endothelial cells, and cutaneous nerves may produce several endogenous pruritogens, including histamine, kinins, proteases, neurotrophins, some opioids, and cytokines [8]. Many of these mediators and modulators released at the periphery can directly activate the itch-sensitive C-fibers by specific receptors on the nerve endings or they can act indirectly by inducing the release of pruritogenic mediators and modulators from other cells [9]. Moreover interactions among them can exacerbate and strengthen itch sensation to promote chronic pruritic diseases [10].

Although the exact pathogenesis of LP is still not fully elucidated, here we would like to discuss some of possible pruritic mediators and mechanisms which may be involved in pruritus present in LP.

## 2. Interleukin 31

LP results from an autoimmune reaction, and it is believed that cell-mediated autoimmunity directed against keratinocytes of basal layer results in the formation of subepithelial infiltrate, composed initially of CD4+ lymphocytes and, subsequently, CD8+ cytotoxic cells. Activated lymphocytes produce a variety of cytokines, and it seems very probable that at least some of these cytokines might also assist in the development of itch in LP. 

 Some previous studies suggested that interleukin 31 (IL-31) and its receptor components IL-31RA and OSMR could be a key cytokine pathway involved in itching which accompanies a number of inflammatory skin conditions, mostly atopic dermatitis [11–13]. IL-31 is a newly discovered, T-cell-derived, short-chain member of the alpha-helical family of IL-6 cytokines. IL-31 receptors were found to be localized in dorsal root ganglia, but itch is rather induced by binding of this cytokine to receptors located on sensory neurons in the skin. Transgenic mice that overexpress IL-31 developed severe pruritus and pruritic skin lesions. In an AD-like murine model (NC/Nga mice), high IL-31 mRNA expression was associated with scratching behavior, while an anti-IL-31 antibody reduced scratching desire [12, 13]. 

 It was also reported that TNF-*α*, a proinflammatory cytokine which plays an important role in the pathogenesis of LP and is elevated in the skin of patients with LP, may stimulate IL-31 expression, a phenomenon that might be responsible for escalation of itch sensation in LP [14, 15]. Ongoing studies should verify the hypothesis, whether IL-31 is indeed a key player involved in the pathogenesis of pruritus in LP and whether therapies directed against this cytokine will provide benefit for treated patients. 

## 3. PAR: Protease Activated Receptors

Elevated production of proinflammatory cytokines leads to increased expression of HLA-DR antigens, intercellular adhesion molecule (ICAM) and Fas receptors which cause apoptosis of keratinocytes. Activated T lymphocytes are attracted to the dermoepidermal junction, where they induce apoptosis of basal layer of keratinocytes; T-cell surface CD95L (Fas ligand) binds to CD95 (Fas) on the keratinocyte surface and activates the caspase cascade resulting in keratinocyte apoptosis [16]. These proteases, mainly of caspase family, play a crucial role in apoptosis, but it was also described that some enzymes released from apoptotic cells may activate protease activated receptors (PAR). 

PAR belongs to the family of G-protein-coupled receptors. Activation of PAR is initiated by the cleavage of the N terminus of the receptor to generate a new tethered ligand terminus, which activates PAR itself. Synthetic peptides which have an amino acid sequence similar to the tethered ligand are also able to activate PAR. Four PAR subtypes PAR-1 to PAR-4, have been identified so far [17, 18]. Tryptase acts on PAR-2 and on PAR-1, but only at high concentration, trypsin on PAR-1, PAR-2, and PAR-4 but not on PAR-3; thrombin acts on PAR-1, PAR-3, and PAR-4 but not on PAR-2, while kallikreins (KLK), mainly KLK5 and KLK14 act only on PAR-2 [18, 19]. Remarkably, PAR-2 has been recently shown to be involved in chronic itch, suggesting that proteases from apoptotic cells may partake in pruritus pathogenesis [17]. One study also reported that mice which overexpressed epidermal KLK7 displayed massive itchy behavior [20]. Another study demonstrated that trypsin-induced scratching behavior in mice was inhibited by a PAR-2 blocking peptide, suggesting the role of serine protease/PAR-2 signaling in pruritus [21, 22]. PAR-2 is reported to interact synergistically with transient receptor potential (TRP) vanilloid type 1 (TRPV1), which belongs to the superfamily of TRP channels, thereby amplifying itch sensation. These findings suggest that serine protease inhibitors or PAR-2 antagonists might be a promising therapeutic tool for the management of itching in the future and could possibly help to break the vicious itch-scratch cycle in pruritic dermatoses [23, 24]. Furthermore, expression of PAR has been shown to be increased in diseases with hypertrophic granular layer such as LP, and it is quite likely that PAR receptors are indeed involved in itch which accompanies LP. 

## 4. Toll-Like Receptors 

Another potential mechanism possibly taking part in pathogenesis of LP is the activation of toll-like receptors (TLRs). TLRs have recently emerged as key sensors of invading microbes, acting through recognition of pathogen-associated molecular patterns (PAMP) [25]. Recognition of ligands by the TLR leads to a series of signaling events resulting in the induction of acute host responses necessary to kill pathogens. In addition to PAMP, TLRs bind endogenous molecules such as heat shock proteins. TLRs are also responsible for the induction of dendritic cell maturation, which is responsible and necessary for the initiation of adaptive immune responses by producing large amounts of various cytokines activating other components of immune system, mainly lymphocytes [26]. 

 Some members of the TLR family are also involved in the pathogenesis of autoimmune and chronic inflammatory response. In addition, TLR7 was described as a novel receptor mediating itch sensation [27]. These receptors were found to be localized in dorsal root ganglia and on sensory neurons in the skin. Interestingly, their agonists relieved pruritus in laboratory experiments. Referring to the participation of TLRs in the pathogenesis of LP, it seems reasonable to evaluate their role as possible itch mediators in this disease. 

## 5. Opioid Receptors 

Some authors suggested a close relationship between LP and emotional stress. Thus, it seems probable that the opioid system in the skin may be another potential player of pruritus in LP. It was supposed that activation of *μ*-opioid receptors induces pruritus, while activation of *κ*-opioid receptors exerts an opposite effect [28]. However, the pathogenesis of opioid-induced itch is still not completely understood, albeit some mechanisms have been proposed. First refer to the influence of opioid system on the production of pruritogens or other cytokines in keratinocytes [29–31]. There have been numerous studies regarding the immune actions of opioids, and immune cells have been identified as their targets. Activation of *κ*-opioid receptors decreases the inflammatory response by downregulating several cytokines and chemokines. Meanwhile, activation of *μ*-opioid receptors may induce a proinflammatory response [28]. Another mechanism of itch sensation is the activation of *μ*- and/or *κ*-opioid receptors directly on sensory neurons [32, 33]. Lately it was reported that some *μ*-opioid receptor-immunoreactive nerve fibers expressed gastrin-releasing peptide, which may be a marker for itch-specific nerves [34, 35]. Future studies should indicate which mechanism is indeed involved in chronic pruritus pathogenesis and whether activation of opioid system results in itch accompanying LP.

## 6. Conclusions

Summarizing, itch is an important and burdensome symptom of LP; however, this symptom has been poorly studied in LP. Pathogenesis of itch in LP is still indifferently understood, and there is no effective therapeutic modalities alleviating pruritus in patients suffering from this disease. We hope that in the near future new studies will be initiated to better characterize and understand itch in LP. We do believe that such studies may help in the development of new effective antipruritic strategies for LP.
